# Factors Associated with Loneliness and Psychological Distress in Older Adults During the COVID-19 Pandemic in Kazakhstan: A Cross-Sectional Study

**DOI:** 10.3390/medicina61040703

**Published:** 2025-04-11

**Authors:** Aigulsum Izekenova, Assel Izekenova, Dinara Sukenova, Dejan Nikolic, Yineng Chen, Alina Rakhmatullina, Ardak Nurbakyt

**Affiliations:** 1 Department of Epidemiology with the Course of HIV, Asfendiyarov Kazakh National Medical University, Almaty 050012, Kazakhstan; izekenova.a@kaznmu.kz; 2 Center for Social and Business Research, Kenzhegali Sagadiyev University of International Business, Almaty 050012, Kazakhstan; izekenova.a@uib.kz (A.I.); alinatatt@gmail.com (A.R.); 3 School of Social Work, Michigan State University, East Lansing, MI 48824, USA; 4 Department of Public Health, Asfendiyarov Kazakh National Medical University, Almaty 050012, Kazakhstan; a.nurbakyt@kaznmu.kz; 5 Faculty of Medicine, University of Belgrade, 11000 Belgrade, Serbia; dejan.nikolic@udk.bg.ac.rs; 6 Department of Physical Medicine and Rehabilitation, University Children’s Hospital, 11000 Belgrade, Serbia; 7 Department of Epidemiology and Biostatistics, College of Integrated Health Science, University at Albany, Albany, NY 12222, USA; ychen77@albany.edu

**Keywords:** sociodemographic factors, health-related factors, loneliness, anxiety, depression, older adults, COVID-19

## Abstract

*Background and Objectives:* In Kazakhstan, during the COVID-19 pandemic, older adults faced unique challenges, such as limited healthcare resources and prolonged periods of social isolation. The aim of our study was to evaluate the sociodemographic and health-related factors associated with loneliness and psychological distress in older adults during the COVID-19 pandemic in Kazakhstan. *Materials and Methods:* In this cross-sectional study, a total of 445 participants aged 60 and above were recruited from Kazakhstan during the COVID-19 pandemic. The Patient Health Questionnaire-4 (PHQ-4) was used to measure psychological distress, anxiety, and depression. Loneliness was assessed using the UCLA Loneliness Scale (UCLA-3). The sociodemographic and health-related variables of the tested participants were analyzed. *Results:* Mean values from the UCLA-3 (*p* < 0.001), PHQ-4 (*p* < 0.001), anxiety (*p* < 0.001), and depression (*p* < 0.001) scores significantly differed between different categories of self-reported overall health. Significantly higher mean values were found in older adults with hypertension for UCLA-3 (*p* = 0.025), PHQ-4 (*p* = 0.001), anxiety (*p* = 0.001), and depression (*p* = 0.017); diabetes for UCLA-3 (*p* = 0.023), PHQ-4 (*p* = 0.029), and depression (*p* = 0.001); chronic heart failure for UCLA-3 (*p* = 0.005), PHQ-4 (*p* < 0.001), anxiety (*p* = 0.001), and depression (*p* < 0.001); cerebrovascular disease for UCLA-3 (*p* = 0.024), PHQ-4 (*p* = 0.002), anxiety (*p* = 0.001), and depression (*p* = 0.027); cardiovascular disease for UCLA-3 (*p* < 0.001), PHQ-4 (*p* < 0.001), anxiety (*p* < 0.001), and depression (*p* < 0.001); dementia for anxiety (*p* = 0.046); being single for UCLA-3 (*p* = 0.009), PHQ-4 (*p* = 0.031), and depression (*p* = 0.028); other ethnic backgrounds for PHQ-4 (*p* = 0.004) and anxiety (*p* = 0.013); and living in an urban place for PHQ-4 (*p* = 0.043). Being single was shown to be a significant predictor for loneliness (OR 2.21; 95%CI 1.28–3.84), anxiety (OR 2.09; 95%CI 1.14–3.84), and depression (OR 4.23; 95%CI 1.95–9.15). Below-average (OR 5.79; 95%CI 1.09–30.90) self-reported overall health was shown to be a significant predictor of anxiety. *Conclusions*: Our study demonstrated that numerous sociodemographic and health-related factors were associated with loneliness, anxiety, and depression in older adults from Kazakhstan during the COVID-19 pandemic.

## 1. Introduction

The COVID-19 pandemic has had devastating effects globally, directly or indirectly affecting all sectors with overburdened health systems in numerous countries [1]. According to a report from 3 October 2023, there were 1,498,668 confirmed cases of COVID-19 and 19,071 resulting deaths in Kazakhstan [2]. Aside from the respiratory illness caused by COVID-19, there was also a significant impact on physical and psychological health [3]. Increased levels of anxiety, depression, psychological distress, and post-traumatic stress disorder were reported among people during the pandemic [4]. It was pointed out that COVID-19 might trigger or exacerbate chronic conditions, including cardiovascular and respiratory diseases as well as neurodegenerative conditions [5]. Aside from direct health effects from the virus, indirect effects were also described, particularly in terms of fear, stigma, depression, and anxiety. Individuals with identified risk factors for severe degrees of COVID-19, such as diabetes, cardiovascular and respiratory diseases, appeared to be more susceptible to these indirect effects [6]. Older adults with one or more comorbidities were determined to have a higher risk of serious complications from COVID-19 [7]. Zhussupov et al., in their study, stated that age and the presence of comorbidities were directly related to the severity of COVID-19 symptoms and lethality in Kazakhstan [8]. Aside from acute-phase COVID-19, long COVID is an emerging condition. The incidence of long COVID in non-hospitalized patients is estimated to be between 10 and 30%; in hospitalized patients, between 50 and 70%; and in vaccinated patients, between 10 and 12% [9].

In a systematic review and meta-analysis by Nam et al., it was reported that older people had lower levels of anxiety, depression, and perceived stress [10]. Moreover, in a systematic review by Ciuffreda et al., it was stated that being female, poor sleep quality, poor motor function, and loneliness were factors associated with anxiety and depression in older adults during the COVID-19 pandemic [11]. Additionally, in a study by Jemal et al., factors significantly associated with anxiety symptoms in older adults during the COVID-19 pandemic included being female, the presence of chronic disease, and poor knowledge of the COVID-19 pandemic; meanwhile, for depression symptoms, additional factors were reported, including being 81 years or older, the presence of chronic disease, and lacking social support [12].

In order to control the spread of COVID-19, social distancing and self-isolation measures were introduced [7]. It was reported that social distancing promotes feelings of loneliness and that social exclusion due to isolation is considered a risk factor for cognitive impairment [13]. In a study by Rodney et al., it was stated that loneliness can lead to cardiovascular disease, depression, cognitive dysfunction, and increased mortality rates [14].

The devastating effects of the COVID-19 pandemic have been documented, showing an increase in unhealthier lifestyle habits, such as reduced physical activity, increased alcohol consumption and tobacco smoking, and worsening eating habits [15].

In Kazakhstan, like in many other countries, older adults faced unique challenges during the pandemic, such as limited healthcare resources and prolonged periods of social isolation. Understanding the factors influencing their quality of life is crucial for designing effective interventions and policies to improve their well-being in the post-pandemic era.

Therefore, our study aimed to evaluate the sociodemographic and health-related factors associated with loneliness and psychological distress in older adults during the COVID-19 pandemic in Kazakhstan.

## 2. Method

### 2.1. Study Design and Participants

In this cross-sectional study, 445 participants aged 60 and older out of 458 individuals were recruited from both urban and rural areas in Kazakhstan. The response rate of the study participants was 97.2%, with 13 individuals excluded due to poor-quality data from the sociological survey. The survey was conducted from June to July 2022 by trained individuals in a face-to-face format. We utilized a stratified sampling technique for our study, which was conducted in the Republic of Kazakhstan, specifically in the city of Almaty and the surrounding Almaty Region. This study group was a representative sample of the Kazakhstan population in terms of gender, age, and place of residence.

In Kazakhstan, the retirement age is 60 and 63 years for females and males, respectively [16]. Therefore, the decision was made to include individuals starting at 60 years in our study.

### 2.2. Inclusion and Exclusion Criteria

The eligibility criteria for participants in this study include the following:

- Adults aged 60 years and above;
- Individuals residing in Almaty City and Almaty Region.

The exclusion criteria for participants in this study include the following:

- Individuals below 60 years of age;
- Individual refusing to provide oral informed consent;
- Individuals with aphasia and hearing loss.

### 2.3. Sampling Methodology and Ethical Considerations

The sampling process was based on the percentage distribution of individuals aged 60 and older in the general population of Almaty City and regional rural areas, as reported by the Bureau of National Statistics of the Republic of Kazakhstan [17]. The sample was designed to reflect the national demographic composition, with an equal division between urban and rural populations (50% each). Additionally, the gender representation was aligned with national statistics, ensuring a distribution of 60% women and 40% men among adults aged 60 and older in the Almaty Region [17].

In this study, confidentiality was a key ethical consideration to ensure the privacy and protection of our participants’ data. A professional team of interviewers carried out the data collection process. Before participation, all older adults were informed about the research objectives, the research team, and their rights, including the option to leave or stop the survey at any time without any consequences. To maintain anonymity, no names were recorded in the dataset. Participants provided oral informed consent before proceeding with the interviews. For clarity and reliability, all interviews were recorded. The collected responses were securely stored in a cloud-based system, with participants assigned unique ID numbers instead of names. Access to the dataset was restricted to the main research team members and was secured with a password to prevent unauthorized access.

### 2.4. Instruments

The Patient Health Questionnaire-4 (PHQ-4) scale was used to measure psychological distress. It comprises four items, with responses rated from 0 to 3, where 0 is “not at all”, 1 is “several days”, 2 is “more than half the days”, and 3 is “nearly every day”. The scores were categorized as none (0–2), mild distress (3–5), moderate distress (6–8), and severe distress (9–12) [18]. Anxiety is defined as the total score ≥ 3 of the first two questions (feeling nervous, anxious, or on edge, and not being able to stop or control worrying) from PHQ-4, while depression is defined as the total score ≥ 3 of the third and fourth questions (feeling down, depressed, or hopeless, and little interest or pleasure in doing things) from PHQ-4 [18].

Loneliness was assessed using the UCLA Loneliness Scale (UCLA-3). This 3-item scale assessed loneliness, with total scores ranging from 3 to 9. It comprises three items, with responses rated from 1 to 3, where 1 is “hardly ever”, 2 is “some of the time”, and 3 is “often”. The cutoff score for loneliness was ≥6 [19]. Therefore, scores were categorized as not feeling lonely (3–5) and feeling lonely (6–9).

The UCLA-3 instrument was previously used in a cross-cultural study of Spanish and Russian populations evaluating the psychological impact of the lockdown during COVID-19 [20], while PHQ-4 was adapted to the Russian language for the general population [21].

### 2.5. Data Collection

Data were collected through structured interviews conducted by trained researchers. The questionnaire included the following:

- Sociodemographic information, where the following variables were analyzed: age (the groups were divided into 60–64 years; 65–69 years; 70–74 years; 75–79 years; and 80 years and above), gender (male and female), education (primary (3–4 grades); incomplete secondary (8–9 grades); secondary (10–11 grades); specialized secondary (technical school, college); higher (bachelor, specialist); postgraduate (master, doctor, PhD, professor, candidate of science, doctor of science; and other)), marital status (single; married; widowed; divorced; and other), number of children whom the participants live with (none; 1–3; 4–6; 7–9; and 10 or more), ethnicity (Kazakh and other), and place of residence (urban and rural).
- Health-related information, where the following variables were analyzed: self-reported overall health (weak; below average; average; good; and very good), hypertension (no; yes), diabetes (no; yes), chronic heart failure (no; yes), cerebrovascular disease (no; yes), cardiovascular disease (no; yes), chronic obstructive pulmonary disease (COPD) (no; yes), dementia (no; yes).

For the purpose of this study, we modified the following variables:

- Age group: 60–69 years, 70–79 years, and 80 years and above;
- Education: Elementary and secondary (Primary, Incomplete secondary, and Secondary), Specialized secondary, and University (Higher, Postgraduate, and other);
- Marital status: Single (Single, Widowed, Divorced, and other) and Married;
- Number of children whom the participants live with: Formulated to “live with children”: no, yes (1 and more children)

### 2.6. Statistical Analysis

The sample size for this study was determined to ensure sufficient statistical power to detect significant relationships between factors affecting the quality of life of older adults, particularly loneliness and anxiety, which play a significant role in well-being. A power analysis was conducted to verify the adequacy of the sample size, assuming a small-to-moderate effect size (f^2^ = 0.10), a significance level of 0.05, and a statistical power of 0.80. The results indicated that a minimum of 300 older adults would be required to achieve reliable and generalizable findings. This sample size was deemed sufficient to capture meaningful associations while maintaining the robustness of statistical analyses.

Categorical variables were presented as whole numbers (N) and percentages (%). Continuous variables were presented as mean values (MV) and standard deviation (SD). To test the normality of data distribution for continuous variables, we carried out the Kolmogorov–Smirnov test (Supplementary Material Table S1). Continuous variables were compared using the Mann–Whitney U test for two samples and the Kruskal–Wallis test for three or more samples. Logistic regression analysis was performed to analyze the association between tested variables with the presence of loneliness measured by UCLA-3 and with the presence of anxiety or depression measured by the PHQ-4 questionnaire. The odds ratio (OR) and 95% confidence intervals (CI) were presented in the multivariate backward stepwise logistic regression analysis. Statistical analysis was performed using IBM SPSS statistical software (SPSS for Windows, version 26.0, SPSS, Chicago, IL, USA). Effect size (r) was calculated, and violin plots in the graphs were created using DATAtab software [22].

For logistic regression analysis, the following models were created:

- Model 1: The referential value is not perceiving loneliness (0) versus perceiving loneliness (1).
- Model 2: The referential value is not perceiving anxiety (0) versus perceiving anxiety (1).
- Model 3: The referential value is not perceiving depression (0) versus perceiving depression (1).

The statistical significance was set at *p* < 0.05.

## 3. Results

The study group’s sociodemographic and health-related characteristics are presented in Table 1. Older adults age between 60 and 69 years (60.4%); those with higher, postgraduate and other education (40.7%); average self-reported overall health (45.2%); married (65.6%); and living with no children (70.8%) were most frequently presented, while females (60.9%); those of Kazakh ethnic background (69.4%); not lonely (UCLA-3) (84.9%); and with no particular distresses (PHQ-4) (76.0%) were more frequently presented. Regarding health-related characteristics, older adults without hypertension (56.6%), diabetes (90.8%), chronic heart failure (85.4%), cerebrovascular disease (96.2%), cardiovascular disease (78.2%), COPD (96.4%), and dementia (99.6%) were more frequently presented. Regarding place of residence, the distribution of participants was similar ((urban (49.9%) and rural (50.1%)).

The mean values of UCLA-3 and PHQ-4 for the tested variables are presented in Table 2. The mean values of UCLA-3 significantly differed between different categories of self-reported overall health (Chi^2^ = 32.47; df = 4; *p* < 0.001), with the highest value found in the weak category and the lowest value found in the good category. Significantly higher mean values of UCLA-3 were found in older adults with hypertension (U = 21520.5; z = −2.24; *p* = 0.025; r = 0.11), diabetes (U = 6622.5; z = −2.28; *p* = 0.023; r = 0.11), chronic heart failure (U = 9579; z = −2.80; *p* = 0.005; r = 0.13), cerebrovascular disease (U = 2551; z = −2.25; *p* = 0.024; r = 0.11), cardiovascular disease (U = 13166; z = −3.57; *p* < 0.001; r = 0.17), and those who were single and elderly (U = 19210; z = −2.62; *p* = 0.009; r = 0.12).

The mean values of PHQ-4 significantly differed between different categories of self-reported overall health (Chi^2^ = 40.35; df = 4; *p* < 0.001), with the highest value found in the below-average category and the lowest value in the very good category. Significantly higher mean values of PHQ-4 were found in older adults with hypertension (U = 20229.5; z = −3.24; *p* = 0.001; r = 0.15), diabetes (U = 6673.5; z = −2.19; *p* = 0.029; r = 0.10), chronic heart failure (U = 8626; z = −3.84; *p* < 0.001; r = 0.18), cerebrovascular disease (U = 2157.5; z = −3.04; *p* = 0.002; r = 0.14), cardiovascular disease (U = 12474.5; z = −4.19; *p* < 0.001; r = 0.20), those who were single and elderly (U = 19739; z = −2.15; *p* = 0.031; r = 0.10), of other ethnic origin (U = 17599; z = −2.91; *p* = 0.004; r = 0.14), and living in urban areas (U = 22184; z = −2.02; *p* = 0.043; r = 0.10).

The mean values for anxiety and depression for the tested variables are presented in Table 3. The mean values for anxiety significantly differed between different categories of self-reported overall health (Chi^2^ = 39.65; df = 2; *p* < 0.001), with the highest value found in the below-average category and the lowest value found in the very good category. Significantly higher mean values for anxiety were found in older adults with hypertension (U = 20413.5; z = −3.23; *p* = 0.001; r = 0.15), chronic heart failure (U = 9190.5; z = −3.34; *p* = 0.001; r = 0.16), cerebrovascular disease (U = 2059.5; z = −3.37; *p* = 0.001; r = 0.16), cardiovascular disease (U = 12546; z = −4.30; *p* < 0.001; r = 0.20), dementia (U = 117; z = −2.00; *p* = 0.046; r = 0.09), and other ethnic backgrounds (U = 18221; z = −2.48; *p* = 0.013; r = 0.12).

Mean values for depression significantly differed between different categories of self-reported overall health (Chi^2^ = 29.38; df = 4; *p* < 0.001), with the highest value found in the below-average category and the lowest value in the good category. Significantly higher mean values for depression were found in older adults with hypertension (U = 21665; z = −2.38; *p* = 0.017; r = 0.11), diabetes (U = 6199.5; z = −3.20; *p* = 0.001; r = 0.15), chronic heart failure (U = 9197; z = −3.62; *p* < 0.001; r = 0.17), cerebrovascular disease (U = 2688; z = −2.21; *p* = 0.027; r = 0.10), cardiovascular disease (U = 13635.5; z = −3.49; *p* < 0.001; r = 0.17), and those who were single and elderly (U = 19987; z = −2.20; *p* = 0.028; r = 0.10).

Logistic regression analysis for the tested variables regarding the presence of loneliness measured by UCLA-3 are presented in Table 4. In multivariate backward stepwise logistic regression analysis, marital status (*p* = 0.005) was significantly associated with loneliness.

Logistic regression for the tested variables regarding the presence of anxiety (Model 2) and depression (Model 3) measured by PHQ-4 is presented in Table 5.

For Model 2, in the multivariate backward stepwise logistic regression model, self-reported overall health (below average (*p* = 0.040)) and marital status (*p* = 0.018) were shown to be significantly associated with anxiety.

For Model 3, in the multivariate backward stepwise logistic regression model, marital status (*p* < 0.001) was shown to be significantly associated with depression.

**Figure 1:**
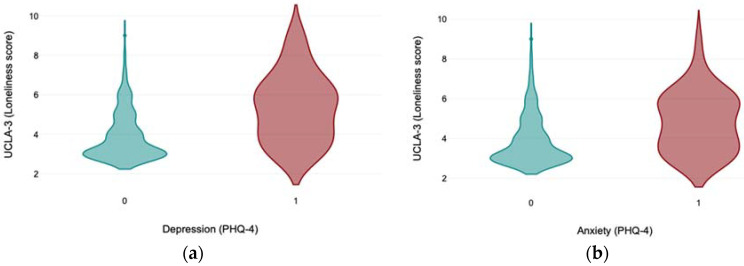
(**a**,**b**) The distribution of loneliness scores measured by UCLA-3 with regard to the presence of depression (**a**) and anxiety (**b**) measured by PHQ-4 in older adults in Kazakhstan during the COVID-19 pandemic (on the X axis, 0 represents the absence of depression or anxiety; 1 represents the presence of depression or anxiety).

Figure 1a,b present the distribution of loneliness scores, measured by UCLA-3, with regard to the presence of depression (a) and anxiety (b), measured by PHQ-4, in older adults in Kazakhstan during the COVID-19 pandemic.

**Figure 2:**
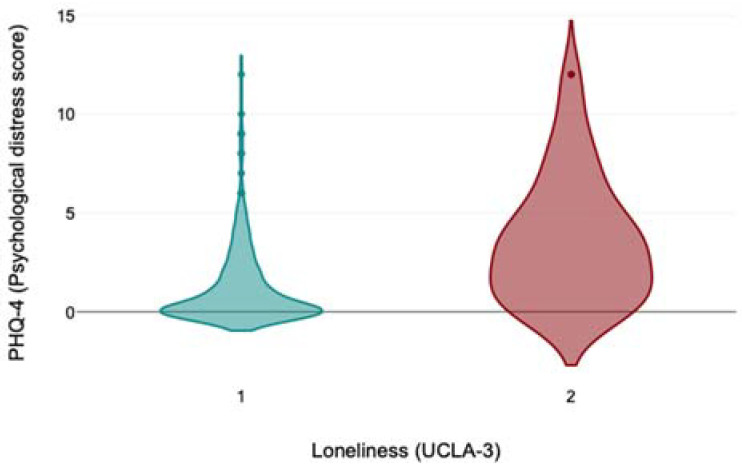
The distribution of psychological distress scores measured by PHQ-4 with regard to the presence of loneliness measured by UCLA-3 in older adults in Kazakhstan during the COVID-19 pandemic (on the X axis, 1 represents the absence of loneliness; 2 represents the presence of loneliness).

Figure 2 presents the distribution of psychological distress scores measured by PHQ-4 with regard to the presence of loneliness measured by UCLA-3 in older adults in Kazakhstan during the COVID-19 pandemic.

## 4. Discussion

The results of our study demonstrate that significantly increased scores for loneliness were found in older adults with lower self-reported overall health, those with hypertension, diabetes, chronic heart failure, cerebrovascular and cardiovascular diseases, as well as those that were single. Furthermore, being single was 2.21 times more likely to lead to perceived loneliness during the COVID-19 pandemic in Kazakhstan. It should be stressed that in our study, the proportion of older adults with diabetes was 9.2%, and in the study by Orazumbekova et al., it was stated that the proportion of people in Kazakhstan with type 2 diabetes mellitus was 8.2–12.5% [23]. Moreover, we found no significant associations between age in older adults during the COVID-19 pandemic and loneliness. This can be partially explained by the fact that individuals over 80 years old were represented in low numbers, and this group has been shown to have a heightened susceptibility to loneliness [24]. Individuals above 80 years of age, compared to middle-aged working people, are almost twice as likely to be lonely [25]. It was stated that social isolation and loneliness in older adults age 50 and above during COVID-19 correlated with cognitive decline [26]. Moreover, in a study conducted in New Zealand, it was shown that older adults who were lonely demonstrated lower physical and mental health scores in comparison to those who were not lonely [27]. In a cross-sectional survey carried out in Canada, it was noted that living alone and being female increased the odds of loneliness in older adults during COVID-19 [28]. However, in another study among Bangladeshi older adults, it was found that being single, those with non-communicable chronic conditions, having poor memory or concentration, and having lower monthly family income increased the odds of loneliness during the COVID-19 pandemic [29]. It was also reported that age, living alone and in rural communities, female gender, lower education, as well as financial status, present risk factors for older adults experiencing loneliness during the first-wave lockdown of COVID-19 [24]. In a study conducted in China, it was found that male gender, middle school level of education and lower, poor knowledge and concerned about acquiring COVID-19, living in a COVID-19 epicenter, and physical health problems are factors that were significantly associated with loneliness in older adults [30]. Our results are, to a certain degree, in line with previous reports, but from the previous findings, it can be seen that in different countries, factors affecting loneliness differed during the COVID-19 pandemic. Several factors could be hypothesized to have potential influence on such pattern including the stage of the COVID-19 pandemic and obtaining more knowledge of preventive measures, modes of transmissions, risk factors for acquiring COVID-19, treatment interventions, social and cultural characteristics in different countries, and health status of individuals, as well as geographical location, including climate. However, it can be postulated that loneliness is, to a certain degree, a complex multidimensional condition that should be approached in multidisciplinary, interdisciplinary, and transdisciplinary ways to determine optimal and effective preventive and interventional measures at all levels of healthcare.

Regarding anxiety and depression, in our study, both scores increased significantly in those with lower self-reported overall health, hypertension, chronic heart failure, cerebrovascular and cardiovascular diseases, while only significantly increased anxiety scores were noticed in those with dementia and those of other ethnic backgrounds. Only significantly increased depression scores were found in those with diabetes and those who were single. Furthermore, in multivariate logistic regression, single participants were 2.09 times more likely to perceive anxiety and 4.23 times more likely to perceive depression, while those with below-average self-reported overall health were 5.79 times more likely to perceive anxiety during the COVID-19 pandemic in Kazakhstan. It was found that psychological distress, which is experienced immediately after a traumatic event or during the traumatic event, is individualized and influenced by numerous factors [31]. In a nationwide survey from China during the COVID-19 pandemic, it was pointed out that significantly higher psychological distress was noticed in females, people aged between 18 and 30 and those above 60 years of life, individuals with higher education, migrant workers, and the middle region of China [32]. In a systematic review and meta-analysis on the predominant general population during the COVID-19 pandemic, it was revealed that females, younger individuals, those living in rural areas, and people with lower socioeconomic status had higher odds for anxiety, while higher odds for depression comprised the same variables except for those in residential areas [33]. A study conducted in Thailand during the COVID-19 pandemic stressed that employment status or financial situation changes, present comorbidities, contact with suspected or known COVID-19 individuals, as well as elevated fear of COVID-19 were factors shown to be associated with an increased psychological distress level [34]. Additionally, female gender, multimorbidity, low education level, and fewer social contacts were found to be associated with increased psychological distress during the first wave of the COVID-19 pandemic in elderly individuals across Europe [35]. Psychological distress can be, to a certain degree, considered a complex phenomenon affected by numerous factors. Different individual, social, and societal characteristics can influence the onset and degree of psychological distress presentation and its evolvement. Therefore, for loneliness, the approach to psychological distress, anxiety, and depression should be multidisciplinary, interdisciplinary, and transdisciplinary in order to achieve optimal and effective preventive and interventional strategies, as well as promote protective measures, bearing in mind an individualized approach to each person.

### 4.1. Study Limitations

Since this study included self-reported data, various forms of biases should be considered, including misunderstanding the proposed measurement to social-desirability bias [36]. This study design is cross-sectional, thus limiting causal relationships. Additionally, the study participants were from one region in Kazakhstan (Almaty City and Almaty Region). Thus, possible sampling limitations and generalizability challenges may exist. Another limitation is the small sample sizes in certain subcategories of selective variables, which may have limited statistical power for analyzing these vulnerable subgroups. Therefore, further studies are needed on larger samples of older adults. Moreover, data collection in this study occurred in mid-2022 (a later phase of the pandemic), thus potentially missing the most acute psychological effects experienced during earlier lockdown periods. In line with this, future studies should include comparative analyses of groups from early and later phases of the COVID-19 pandemic on loneliness and psychological distress. This will provide more information about the potential role of the COVID-19 pandemic, its changes during the different pandemic phases, and the influence of numerous sociodemographic and health-related factors across different phases on the expression and severity degree of loneliness and psychological distress. Moreover, bidirectional causative effects between these factors and loneliness and psychological distress in older adults could be further explored in longitudinal studies for individuals previously included in observational studies during the COVID-19 pandemic and in the post-pandemic period.

### 4.2. Policy and Practice Implications

To improve preparedness for future pandemics, policies should prioritize the healthcare needs of older adults, ensuring they have access to essential medical services and support. Strengthening geriatric healthcare, including home-based and mobile health programs, will help address the vulnerabilities of this population. Mental health services should be integrated into emergency response plans to reduce loneliness and psychological distress, with community-based initiatives and peer support programs playing a key role. Training healthcare professionals in gerontology and chronic disease management will enhance the healthcare system’s ability to respond effectively to aging-related health challenges. Additionally, ensuring equitable access to healthcare through data-driven, multidisciplinary strategies will be crucial for mitigating the impact of future public health crises on older populations.

## 5. Conclusions

Being a single older adult in Kazakhstan during the COVID-19 pandemic was a predictor of perceiving loneliness, anxiety, and depression, while below-average self-reported overall health was a predictor of anxiety.

The complex influence of numerous sociodemographic and health-related factors on loneliness, anxiety, and depression of older adults during the COVID-19 pandemic points to the necessity to create, promote, and implement effective interventional measures on all levels of healthcare and social care.

## Figures and Tables

**Table 1:** The study group’s sociodemographic and health-related characteristics.

Variables		N (%)
Age	60–69 years	269 (60.4)
	70–79 years	146 (32.8)
	80 years and above	30 (6.7)
Gender	Male	174 (39.1)
	Female	271 (60.9)
Education	Elementary and secondary	118 (26.5)
	Specialized secondary	146 (32.8)
	University	181 (40.7)
Self-reported overall health	Weak	13 (2.9)
	Below average	25 (5.6)
	Average	201 (45.2)
	Good	178 (40.0)
	Very good	28 (6.3)
Hypertension	No	252 (56.6)
	Yes	193 (43.4)
Diabetes	No	404 (90.8)
	Yes	41 (9.2)
Chronic heart failure	No	382 (85.8)
	Yes	63 (14.2)
Cerebrovascular disease	No	428 (96.2)
	Yes	17 (3.8)
Cardiovascular disease	No	348 (78.2)
	Yes	97 (21.8)
COPD	No	429 (96.4)
	Yes	16 (3.6)
Dementia	No	443 (99.6)
	Yes	2 (0.5)
Marital status	Single	153 (34.4)
	Married	292 (65.6)
Live with children	No	315 (70.8)
	Yes	130 (29.2)
Ethnic background	Kazakh	309 (69.4)
	Other	136 (30.6)
Place of residence	Urban	222 (49.9)
	Rural	223 (50.1)
UCLA-3	Not lonely	378 (84.9)
	Lonely	67 (15.1)
PHQ-4	None	338 (76.0)
	Mild distress	78 (17.5)
	Moderate distress	18 (4.0)
	Severe distress	11 (2.5)
PHQ-4	Anxiety (yes)	52 (11.7)
	Depression (yes)	35 (7.9)

**Table 2:** Mean values of UCLA-3 and PHQ-4 for tested variables.

Variables		UCLA-3		PHQ-4	
		MV ± SD	*p*	MV ± SD	*p*
Age	60–69 years	3.94 ± 1.24	0.488	1.39 ± 2.14	0.139
	70–79 years	4.12 ± 1.42		1.88 ± 2.60	
	80 years and above	4.07 ± 1.26		1.60 ± 1.96	
Gender	Male	3.95 ± 1.37	0.119	1.41 ± 2.17	0.235
	Female	4.05 ± 1.26		1.66 ± 2.37	
Education	Elementary and secondary	4.14 ± 1.41	0.436	1.61 ± 2.45	0.995
	Specialized secondary	4.01 ± 1.25		1.52 ± 2.08	
	University	3.93 ± 1.27		1.57 ± 2.36	
Self-reported overall health	Weak	5.23 ± 2.31	<0.001	2.85 ± 2.44	<0.001
	Below average	4.56 ± 1.56		3.84 ± 3.30	
	Average	4.26 ± 1.38		1.78 ± 2.29	
	Good	3.60 ± 0.92		1.02 ± 1.94	
	Very good	3.75 ± 1.00		0.89 ± 1.52	
Hypertension	No	3.88 ± 1.22	0.025	1.30 ± 2.12	0.001
	Yes	4.17 ± 1.39		1.92 ± 2.47	
Diabetes	No	3.97 ± 1.28	0.023	1.47 ± 2.21	0.029
	Yes	4.41 ± 1.47		2.49 ± 2.85	
Chronic heart failure	No	3.95 ± 1.29	0.005	1.43 ± 2.25	<0.001
	Yes	4.37 ± 1.34		2.38 ± 2.40	
Cerebrovascular disease	No	3.99 ± 1.29	0.024	1.50 ± 2.22	0.002
	Yes	4.59 ± 1.46		3.29 ± 3.26	
Cardiovascular disease	No	3.90 ± 1.26	<0.001	1.38 ± 2.22	<0.001
	Yes	4.39 ± 1.39		2.25 ± 2.42	
COPD	No	4.00 ± 1.30	0.493	1.55 ± 2.29	0.161
	Yes	4.25 ± 1.48		2.13 ± 2.33	
Dementia	No	4.00 ± 1.30	0.071	1.56 ± 2.29	0.080
	Yes	5.50 ± 0.71		3.50 ± 0.71	
Marital status	Single	4.32 ± 1.58	0.009	2.13 ± 2.91	0.031
	Married	3.85 ± 1.10		1.27 ± 1.83	
Live with children	No	3.98 ± 1.34	0.159	1.65 ± 2.41	0.719
	Yes	4.08 ± 1.21		1.35 ± 1.98	
Ethnic background	Kazakh	3.98 ± 1.33	0.159	1.40 ± 2.24	0.004
	Other	4.07 ± 1.23		1.95 ± 2.37	
Place of residence	Urban	3.96 ± 1.31	0.339	1.70 ± 2.20	0.043
	Rural	4.05 ± 1.29		1.43 ± 2.38	

**Table 3:** Mean values for anxiety and depression measured by PHQ-4 for tested variables.

Variables		Anxiety		Depression	
		MV ± SD	*p*	MV ± SD	*p*
Age	60–69 years	0.79 ± 1.24	0.109	0.60 ± 1.11	0.617
	70–79 years	1.13 ± 1.57		0.75 ± 1.28	
	80 years and above	0.90 ± 1.35		0.70 ± 1.24	
Gender	Male	0.86 ± 1.33	0.497	0.56 ± 1.06	0.202
	Female	0.94 ± 1.40		0.72 ± 1.24	
Education	Elementary and secondary	0.95 ± 1.44	0.923	0.66 ± 1.20	0.699
	Specialized secondary	0.92 ± 1.35		0.60 ± 1.09	
	University	0.87 ± 1.34		0.70 ± 1.24	
Self-reported overall health	Weak	1.77 ± 1.59	<0.001	1.08 ± 1.61	<0.001
	Below average	2.12 ± 1.86		1.72 ± 1.77	
	Average	1.04 ± 1.40		0.74 ± 1.15	
	Good	0.61 ± 1.16		0.41 ± 0.99	
	Very good	0.39 ± 0.88		0.50 ± 1.00	
Hypertension	No	0.74 ± 1.25	0.001	0.56 ± 1.10	0.017
	Yes	1.12 ± 1.49		0.79 ± 1.27	
Diabetes	No	0.86 ± 1.32	0.151	0.61 ± 1.15	0.001
	Yes	1.34 ± 1.77		1.15 ± 1.33	
Chronic heart failure	No	0.84 ± 1.35	0.001	0.59 ± 1.15	<0.001
	Yes	1.33 ± 1.41		1.05 ± 1.29	
Cerebrovascular disease	No	0.87 ± 1.35	0.001	0.63 ± 1.14	0.027
	Yes	1.88 ± 1.65		1.41 ± 1.77	
Cardiovascular disease	No	0.80 ± 1.33	<0.001	0.58 ± 1.14	<0.001
	Yes	1.31 ± 1.43		0.94 ± 1.27	
COPD	No	0.90 ± 1.37	0.217	0.65 ± 1.18	0.103
	Yes	1.13 ± 1.31		1.00 ± 1.15	
Dementia	No	0.90 ± 1.37	0.046	0.66 ± 1.18	0.151
	Yes	2.50 ± 0.71		1.00 ± 0.00	
Marital status	Single	1.16 ± 1.64	0.055	0.97 ± 1.56	0.028
	Married	0.77 ± 1.19		0.50 ± 0.88	
Live with children	No	0.96 ± 1.43	0.411	0.70 ± 1.23	0.659
	Yes	0.78 ± 1.22		0.57 ± 1.03	
Ethnic background	Kazakh	0.81 ± 1.32	0.013	0.59 ± 1.12	0.066
	Other	1.13 ± 1.46		0.82 ± 1.28	
Place of residence	Urban	0.96 ± 1.33	0.081	0.74 ± 1.20	0.112
	Rural	0.85 ± 1.41		0.58 ± 1.16	

**Table 4:** Logistic regression for tested variables regarding the presence of loneliness measured by UCLA-3 (Model 1).

Model 1 (Loneliness)				
Multivariate Backward Stepwise Logistic Regression				
Variables	Categories	OR	95% CI	*p*
Self-reported overall health (Very good, Ref.)	Weak	5.89	0.93–37.42	0.060
	Below average	4.74	0.88–25.58	0.071
	Average	2.96	0.67–13.107	0.153
	Good	0.69	0.14–3.37	0.647
Marital status (Married, Ref.)	Single	2.21	1.28–3.84	0.005

**Table 5:** Logistic regression for tested variables regarding the presence of anxiety (Model 2) and depression (Model 3) measured by PHQ-4.

PHQ-4		Model 2 (Anxiety)			Model 3 (Depression)		
Multivariate Backward Stepwise Logistic Regression							
Variables	Categories	OR	95% CI	*p*	OR	95% CI	*p*
Self-reported overall health (Very good, Ref.)	Weak	6.02	0.95–38.26	0.057	1.64	0.21–12.85	0.638
	Below average	5.79	1.09–30.90	0.040	1.99	0.33–11.92	0.453
	Average	1.52	0.33–6.87	0.590	0.91	0.19–4.31	0.901
	Good	0.85	0.18–4.05	0.837	0.29	0.51–1.61	0.155
Diabetes (No, Ref.)	Yes	-	-	-	2.41	0.93–6.29	0.072
Marital status (Married, Ref.)	Single	2.09	1.14–3.84	0.018	4.23	1.95–9.15	<0.001

## Data Availability

Data are available upon reasonable request from the corresponding author.

## Supplementary Materials

The following supporting information can be downloaded at https://www.mdpi.com/article/10.3390/medicina61040703/s1. Table S1: Results of Kolmogorov-Smirnov test.
