# Supplementary material for: Factors Associated with Loneliness and Psychological Distress in Older Adults During the COVID-19 Pandemic in Kazakhstan: A Cross-Sectional Study
Source: Medicina (Kaunas). 2025 Apr 11;61(4):703. doi: 10.3390/medicina61040703 (PMC12028759; doi:10.3390/medicina61040703)
Supplement: Supplementary file 1 [file medicina-61-00703-s001.zip › medicina-3581960-supplementary.pdf]

Supplementary Table S1. Results of Kolmogorov-Smirnov test

**Variables**		**UCLA-3**	**PHQ-4**	**Anxiety**	**Depression**
		p	p	p	p
**Age**	60-69 years	<0.001	<0.001	<0.001	<0.001
	70-79 years	<0.001	<0.001	<0.001	<0.001
	80 years and above	<0.001	<0.001	<0.001	<0.001
**Gender**	Male	<0.001	<0.001	<0.001	<0.001
	Female	<0.001	<0.001	<0.001	<0.001
**Education**	Elementary and secondary	<0.001	<0.001	<0.001	<0.001
	Specialized secondary	<0.001	<0.001	<0.001	<0.001
	University	<0.001	<0.001	<0.001	<0.001
**Self-reported overall health**	Weak	0.038	0.200	0.024	<0.001
	Below average	0.010	0.087	0.075	0.013
	Average	<0.001	<0.001	<0.001	<0.001
	Good	<0.001	<0.001	<0.001	<0.001
	Very good	<0.001	<0.001	<0.001	<0.001
**Hypertension**	No	<0.001	<0.001	<0.001	<0.001
	Yes	<0.001	<0.001	<0.001	<0.001
**Diabetes**	No	<0.001	<0.001	<0.001	<0.001
	Yes	<0.001	<0.001	<0.001	<0.001
**Chronic heart failure**	No	<0.001	<0.001	<0.001	<0.001
	Yes	<0.001	<0.001	<0.001	<0.001
**Cerebrovascular disease**	No	<0.001	<0.001	<0.001	<0.001
	Yes	0.008	0.012	0.012	0.004
**Cardiovascular disease**	No	<0.001	<0.001	<0.001	<0.001
	Yes	<0.001	<0.001	<0.001	<0.001
**COPD**	No	<0.001	<0.001	<0.001	<0.001
	Yes	0.016	0.010	0.029	<0.001
**Dementia**	No	<0.001	<0.001	<0.001	<0.001
	Yes	-	-	-	-
**Marital status**	Single	<0.001	<0.001	<0.001	<0.001
	Married	<0.001	<0.001	<0.001	<0.001
**Live with children**	No	<0.001	<0.001	<0.001	<0.001
	Yes	<0.001	<0.001	<0.001	<0.001
**Ethnic background**	Kazakh	<0.001	<0.001	<0.001	<0.001
	Other	<0.001	<0.001	<0.001	<0.001
**Place of residence**	Urban	<0.001	<0.001	<0.001	<0.001
	Rural	<0.001	<0.001	<0.001	<0.001
